# Supplementary figures and images for: Processing of the ribosomal ubiquitin-like fusion protein FUBI-eS30/FAU is required for 40S maturation and depends on USP36
Source: eLife. 2021 Jul 28;10:e70560. doi: 10.7554/eLife.70560 (PMC8354635; doi:10.7554/eLife.70560)

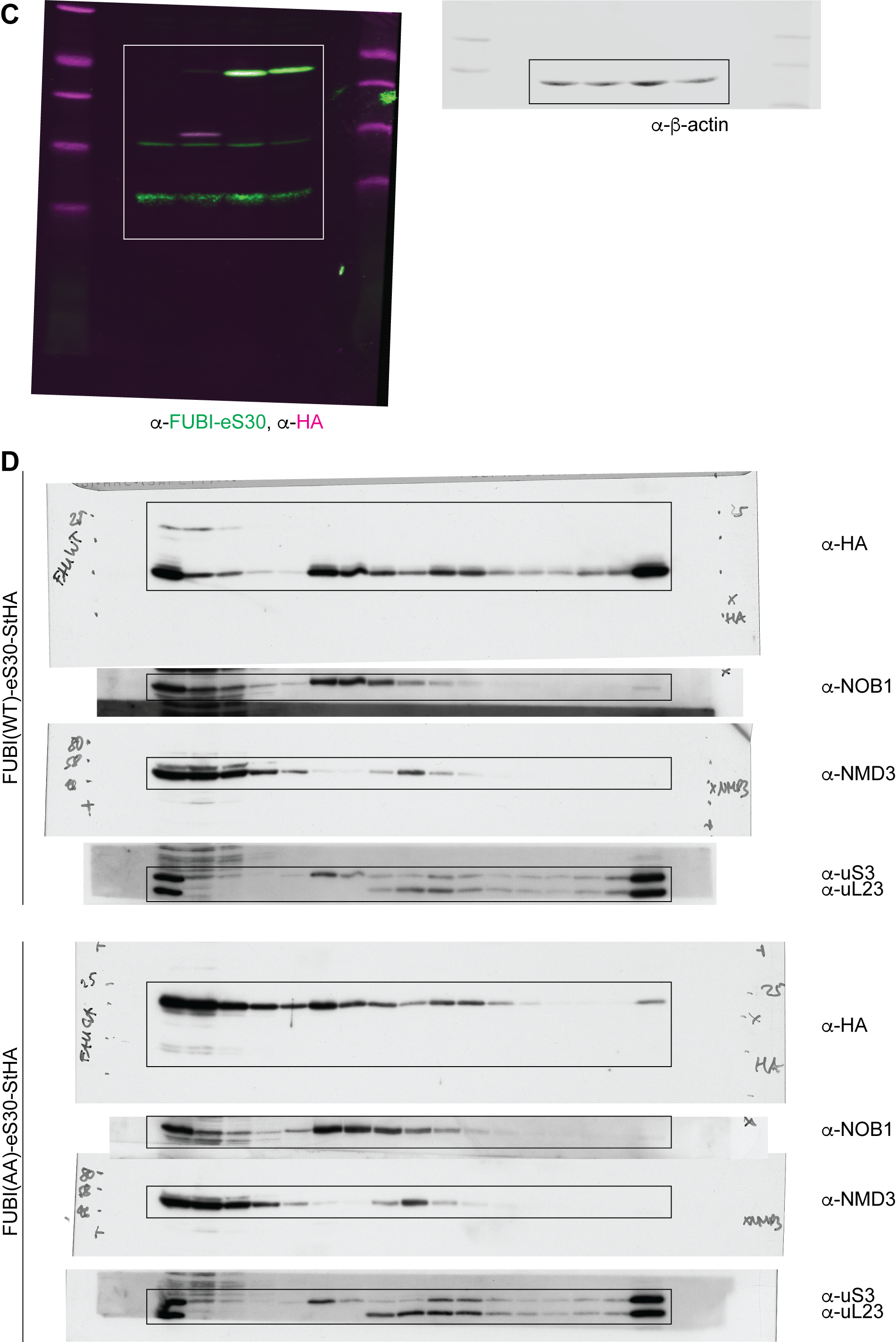

Supplement: Figure 1—source data 1. [file elife-70560-fig1-data1.png.zip › Figure1-sourcedata1.png]

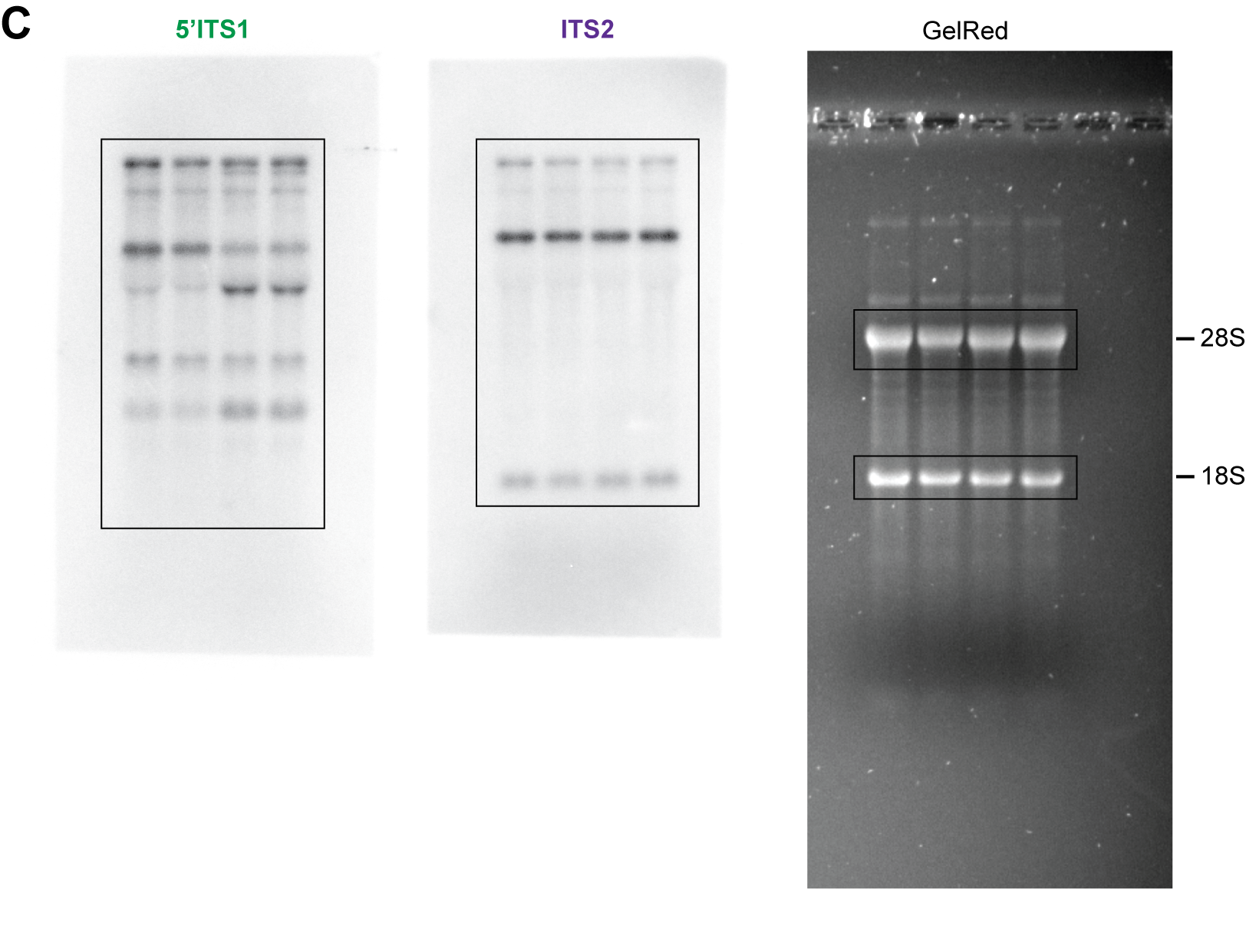

Supplement: Figure 2—source data 1. [file elife-70560-fig2-data1.png.zip › Figure2-sourcedata1.png]

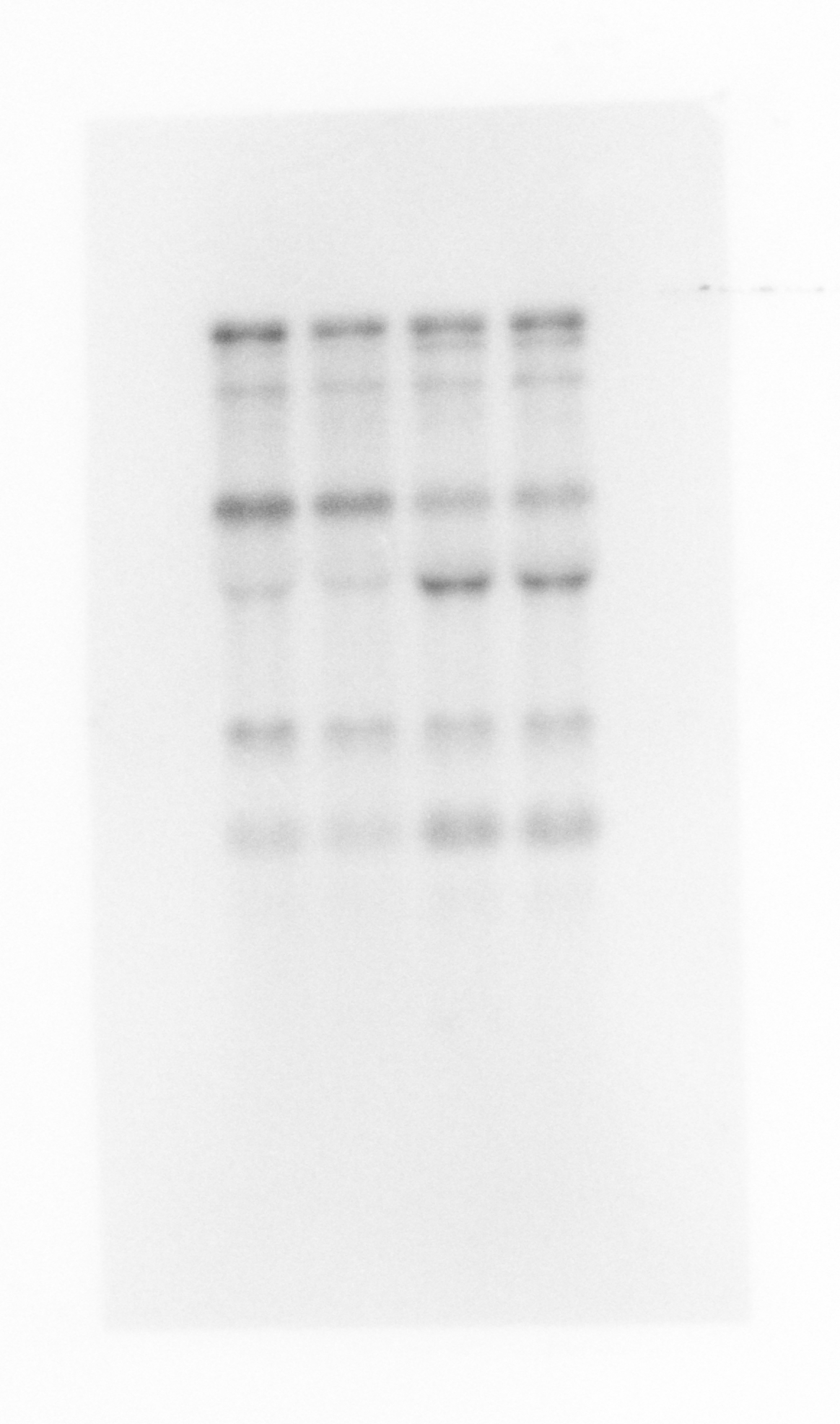

Supplement: Figure 2—source data 2. [file elife-70560-fig2-data2.tiff.zip › Figure2-sourcedata2.tiff]

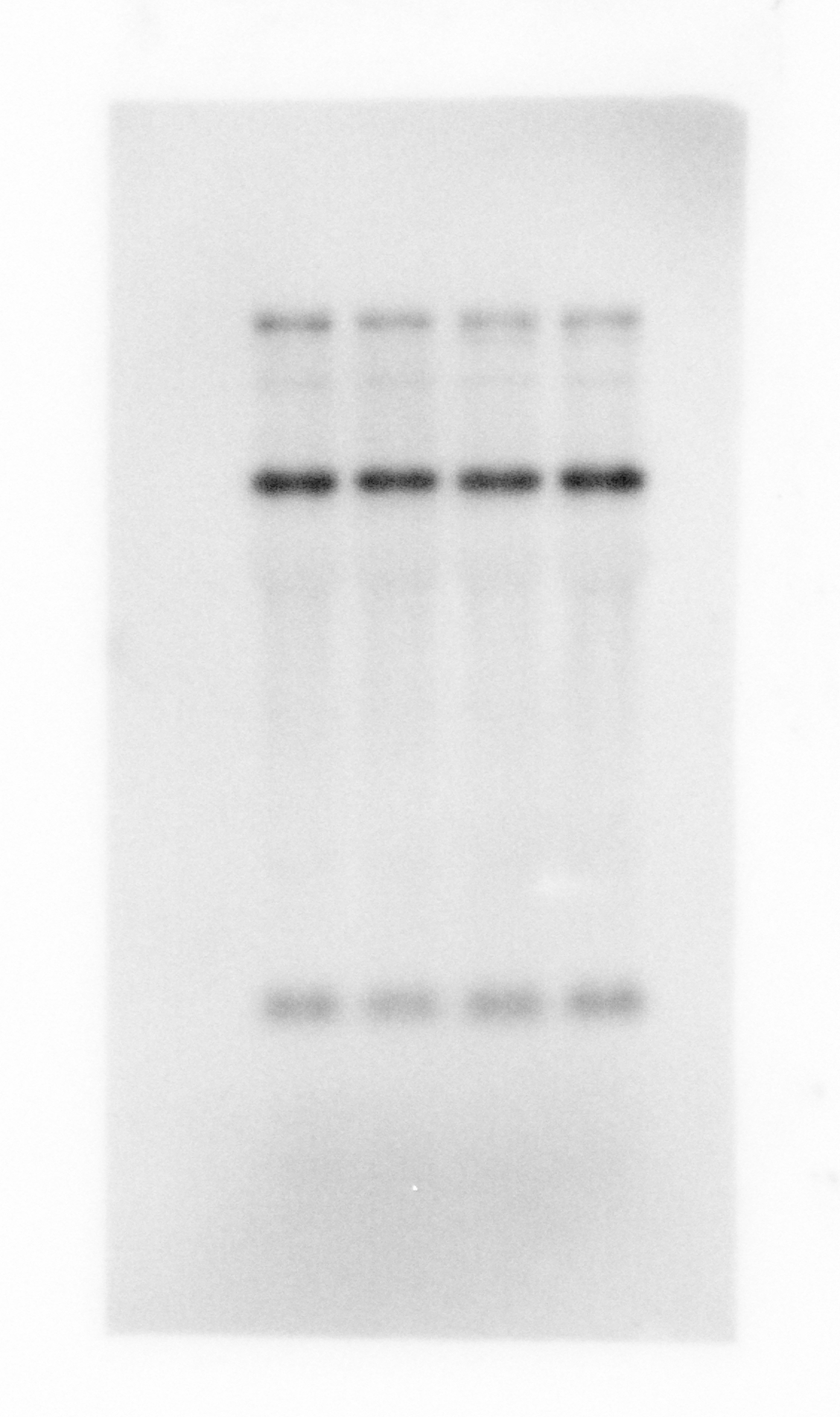

Supplement: Figure 2—source data 3. [file elife-70560-fig2-data3.tiff.zip › Figure2-sourcedata3.tiff]

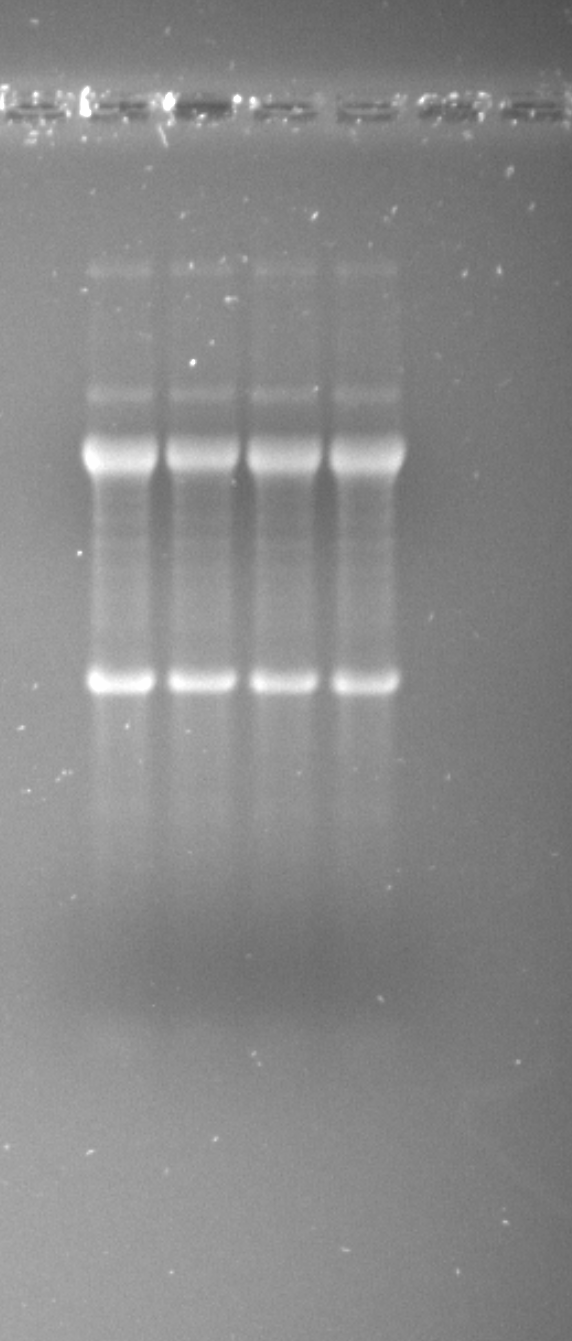

Supplement: Figure 2—source data 4. [file elife-70560-fig2-data4.tif.zip › Figure2-sourcedata4.tif]

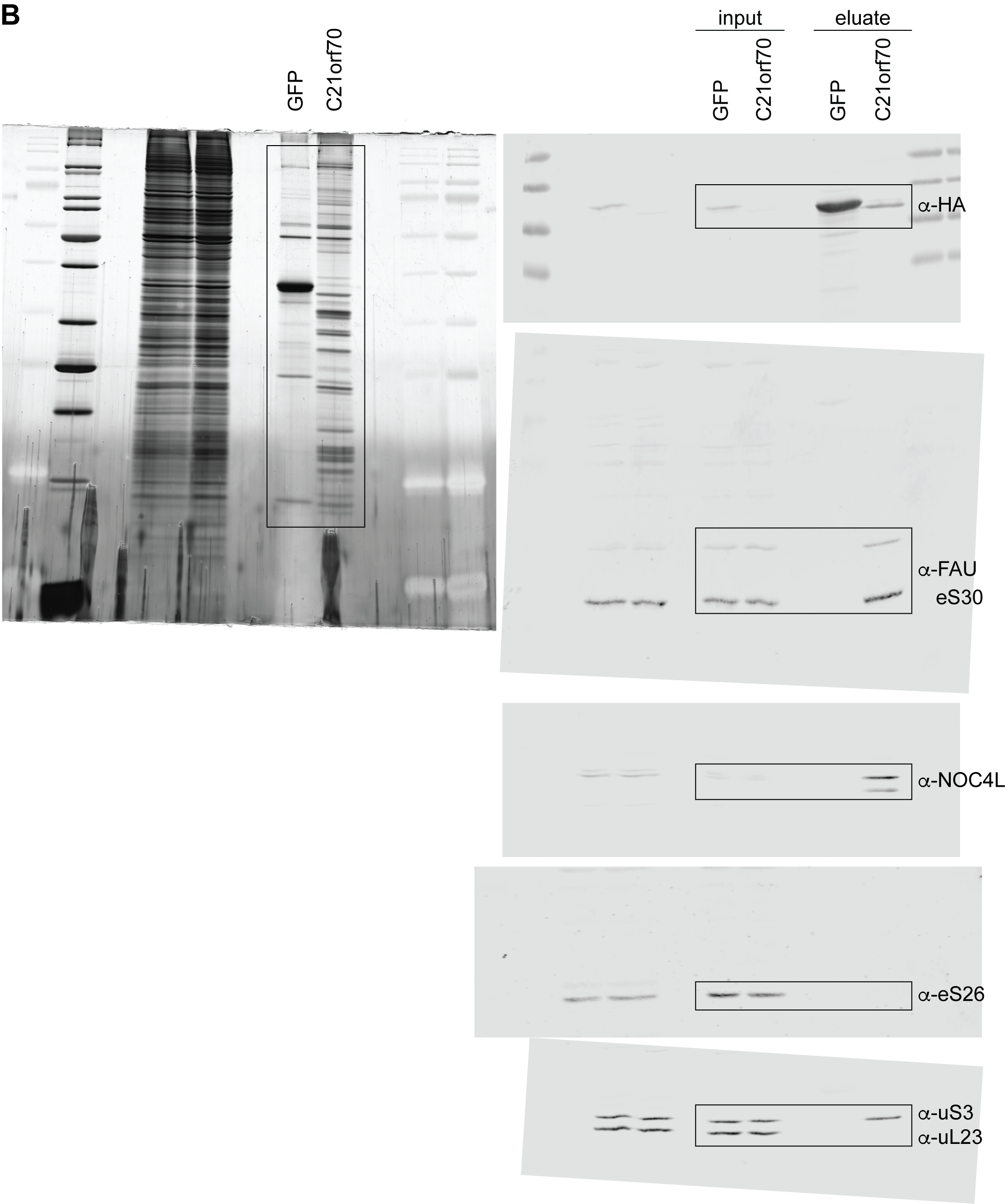

Supplement: Figure 3—source data 1. [file elife-70560-fig3-data1.png.zip › Figure3-sourcedata1.png]

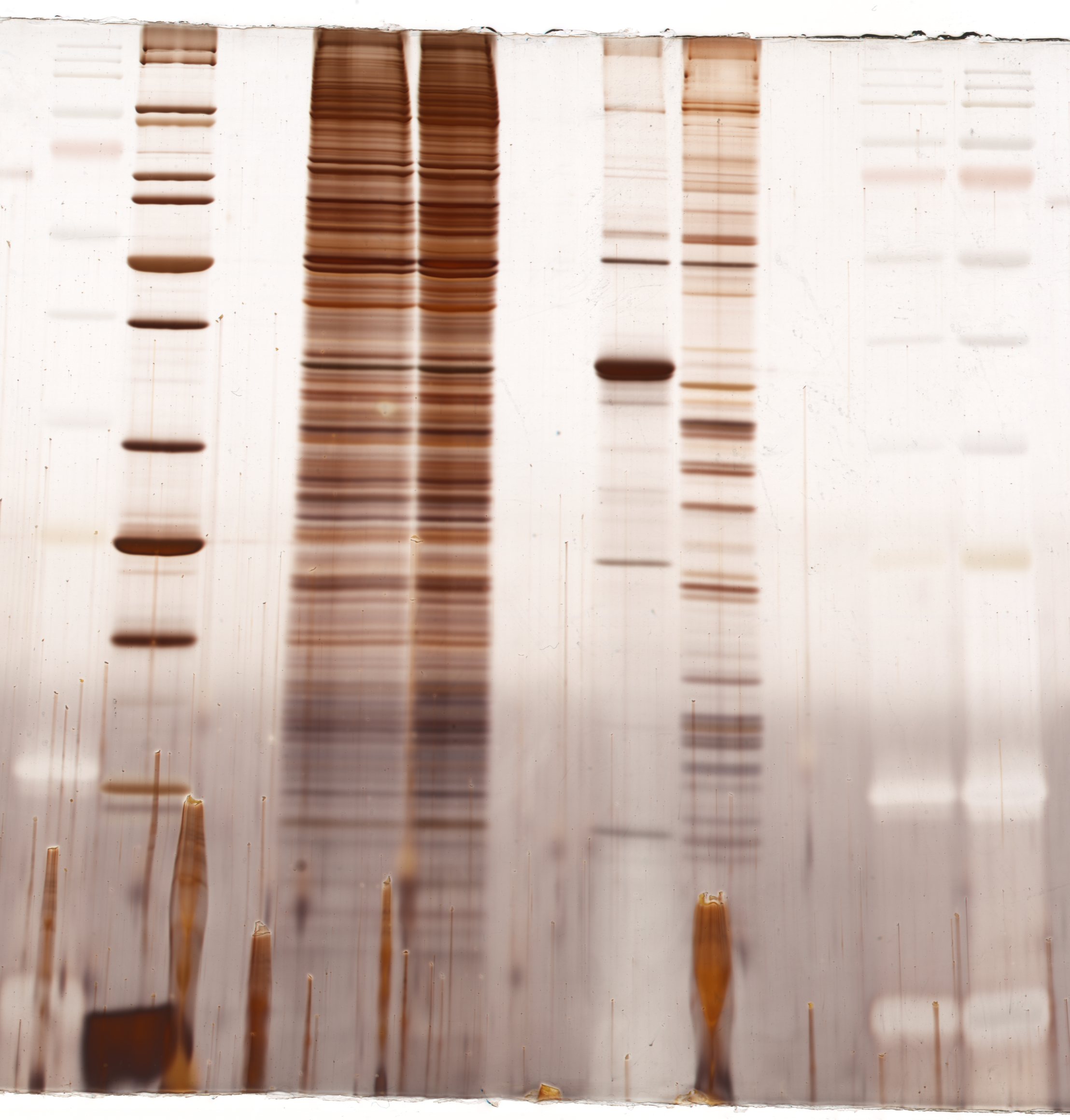

Supplement: Figure 3—source data 2. [file elife-70560-fig3-data2.tif.zip › Figure3-sourcedata2.tif]

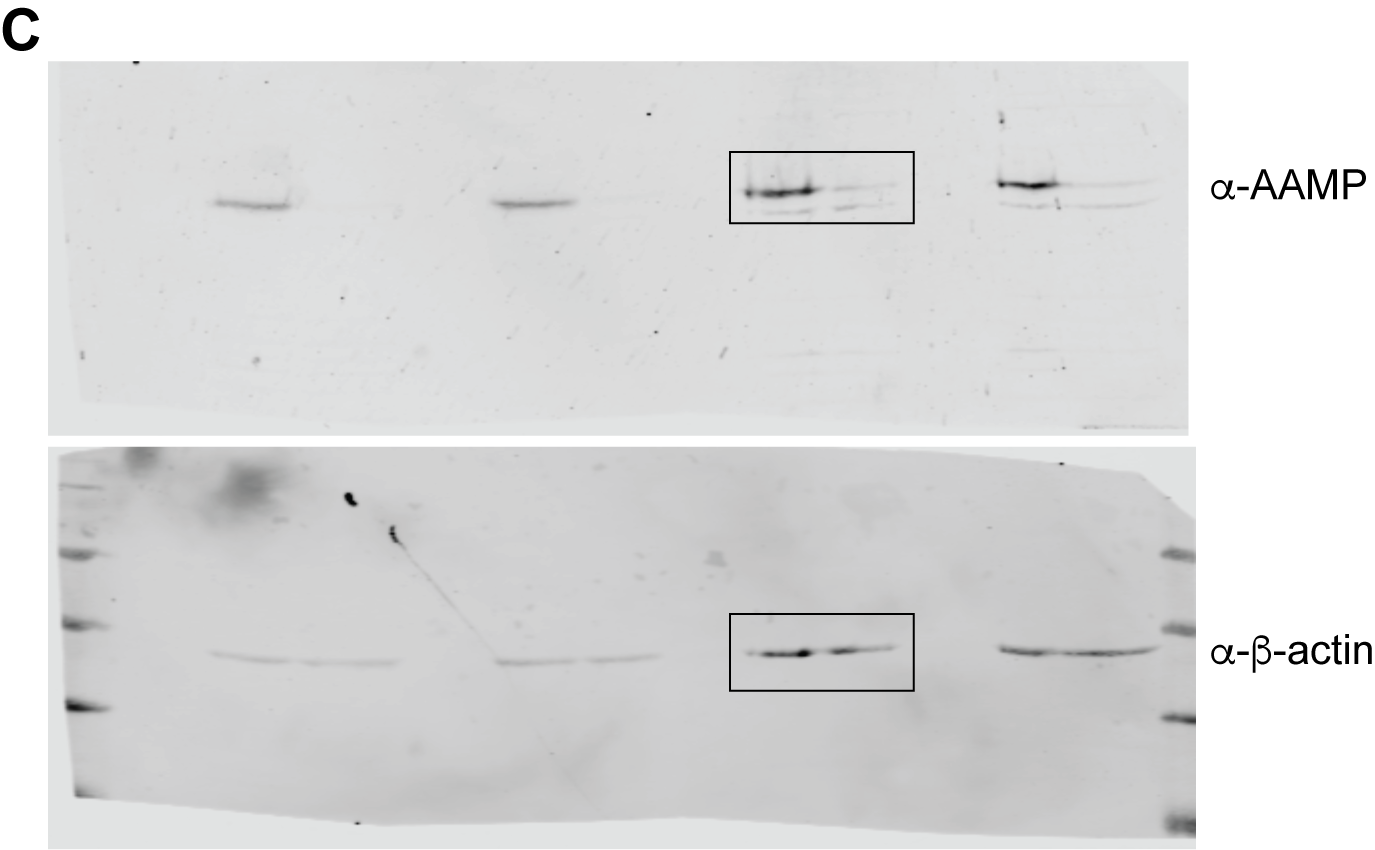

Supplement: Figure 4—figure supplement 1—source data 1. [file elife-70560-fig4-figsupp1-data1.png.zip › Figure4-figuresupplement1-sourcedata1.png]

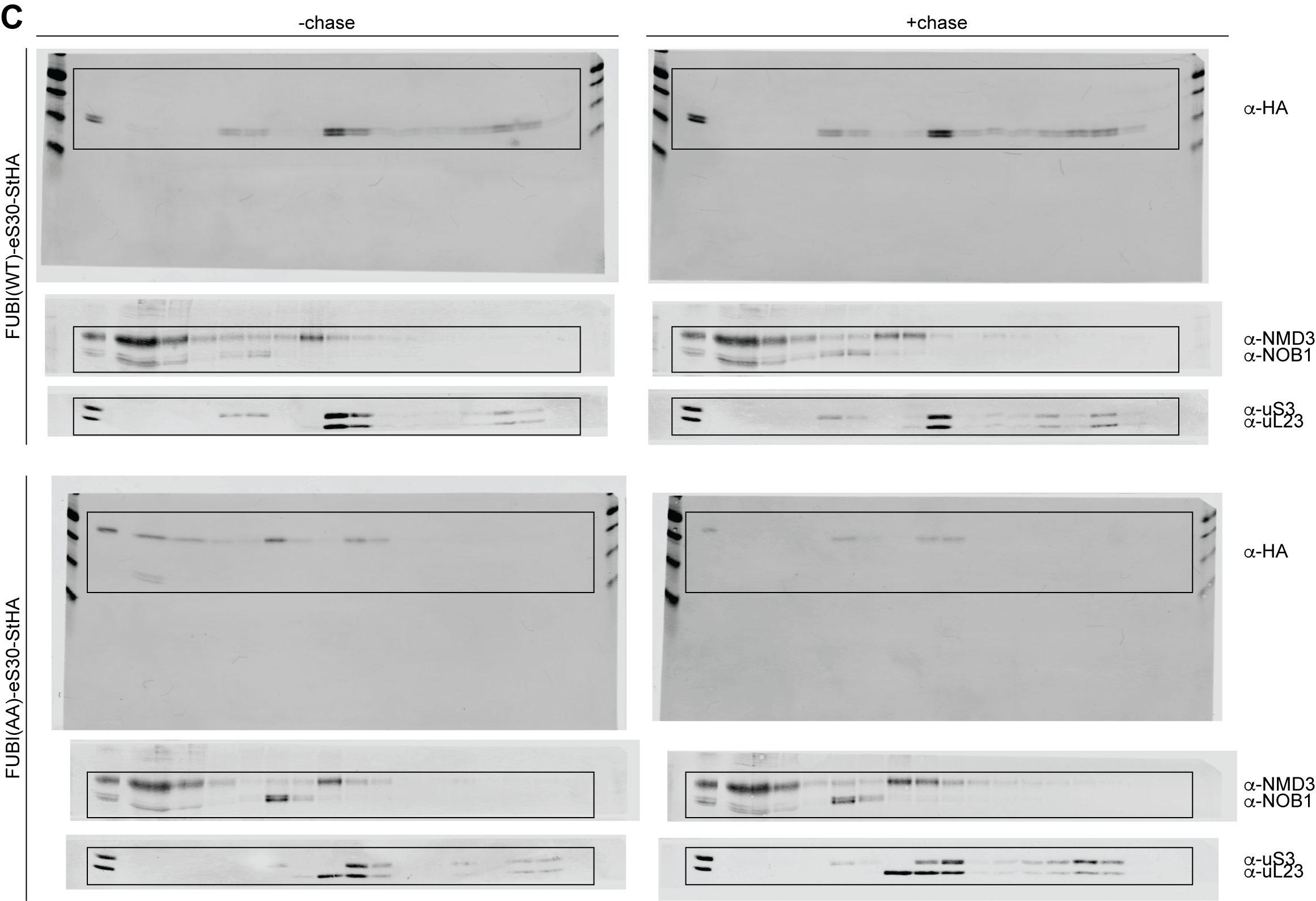

Supplement: Figure 4—figure supplement 2—source data 1. [file elife-70560-fig4-figsupp2-data1.png.zip › Figure4-figuresupplement2-sourcedata1.png]

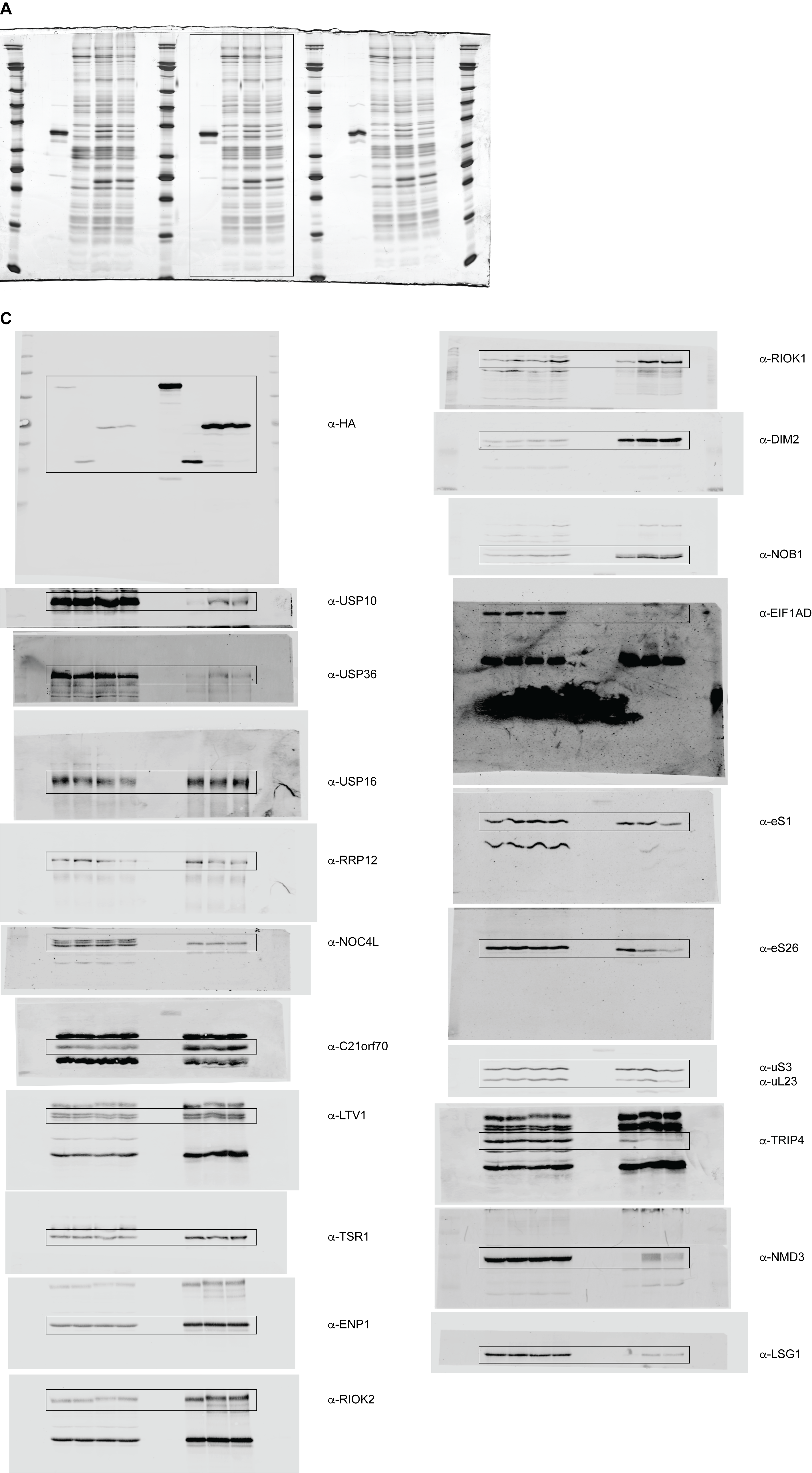

Supplement: Figure 5—source data 1. [file elife-70560-fig5-data1.png.zip › Figure5-sourcedata1.png]

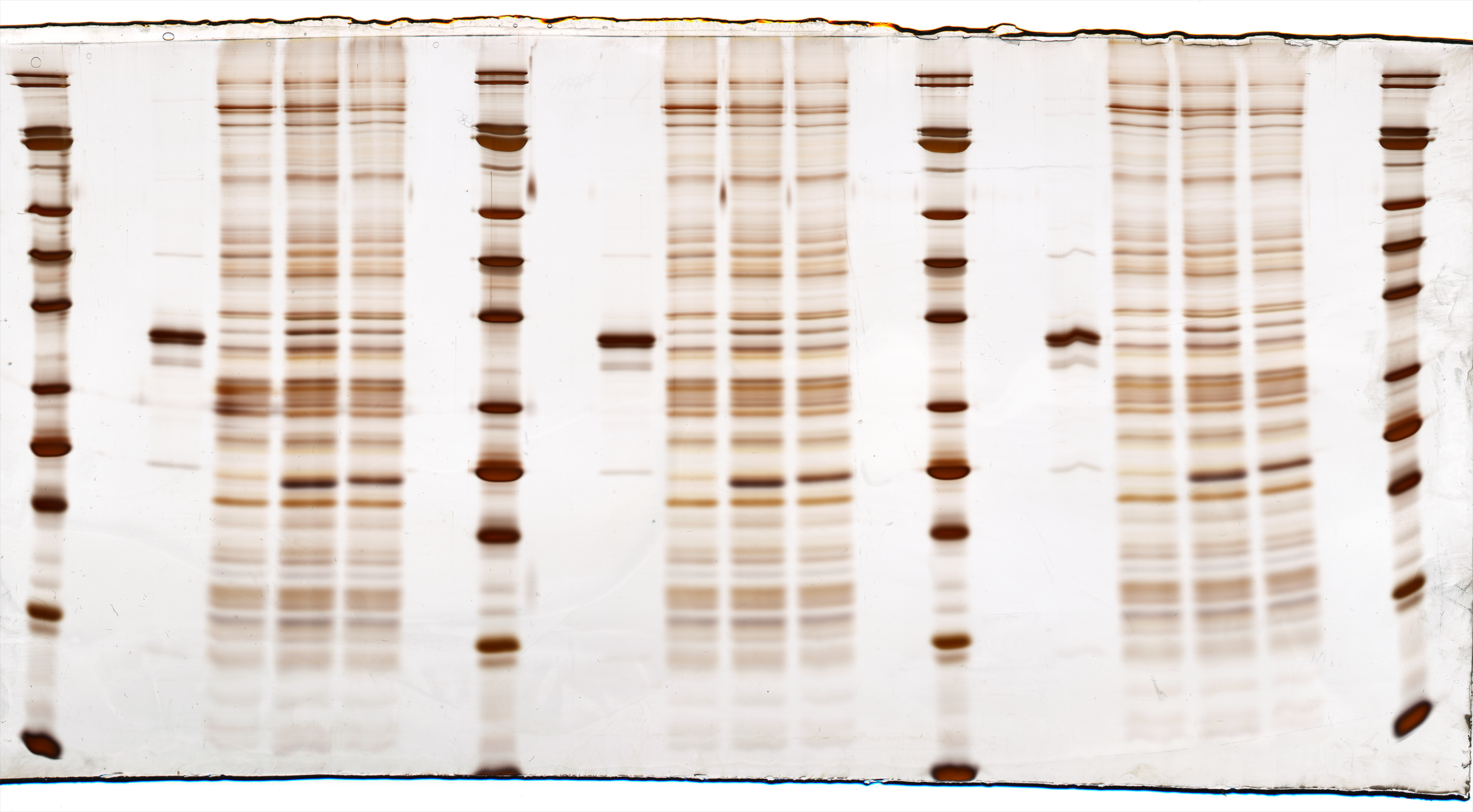

Supplement: Figure 5—source data 2. [file elife-70560-fig5-data2.tif.zip › Figure5-sourcedata2.tif]

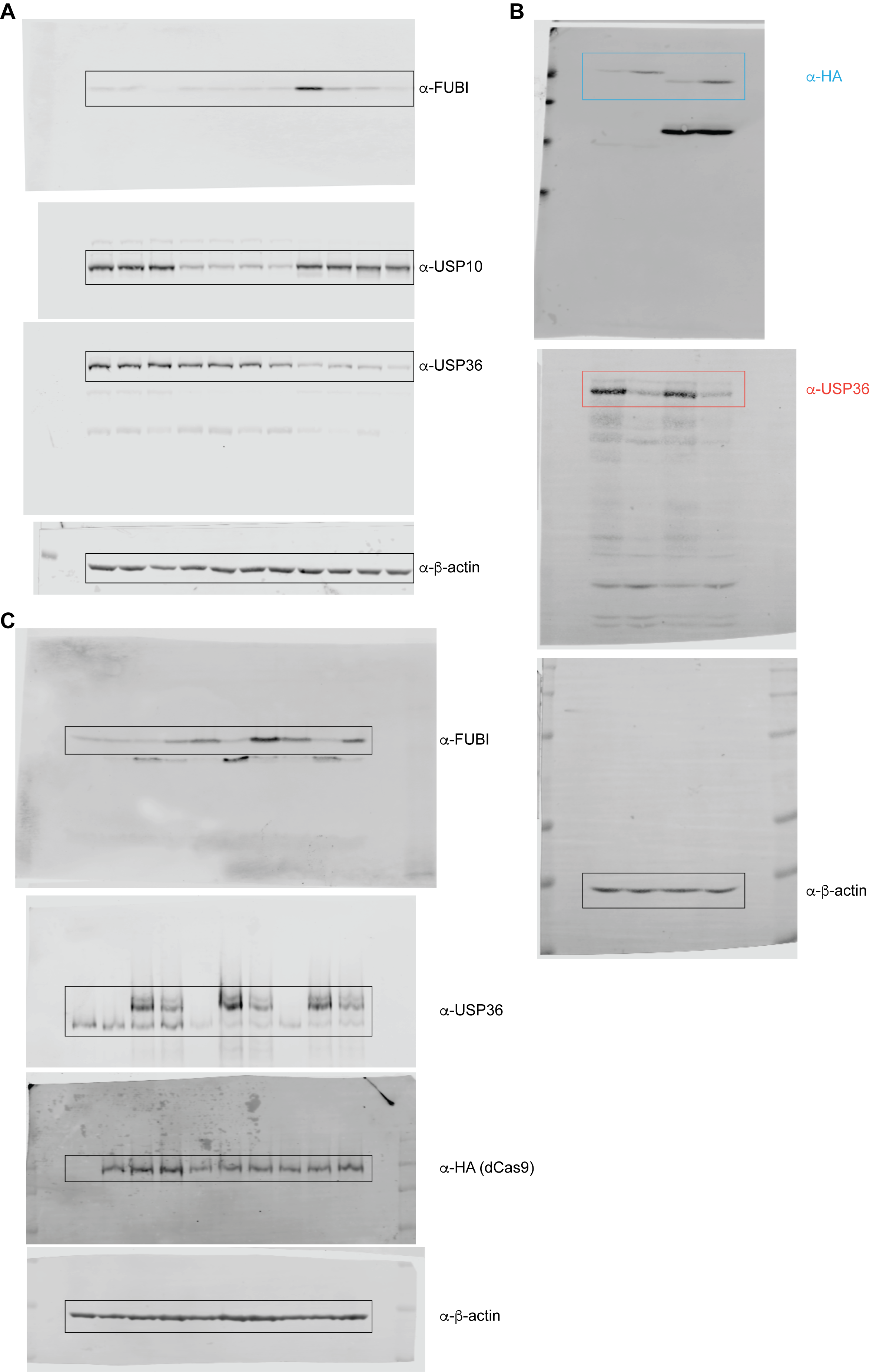

Supplement: Figure 6—source data 1. [file elife-70560-fig6-data1.png.zip › Figure6-sourcedata1.png]

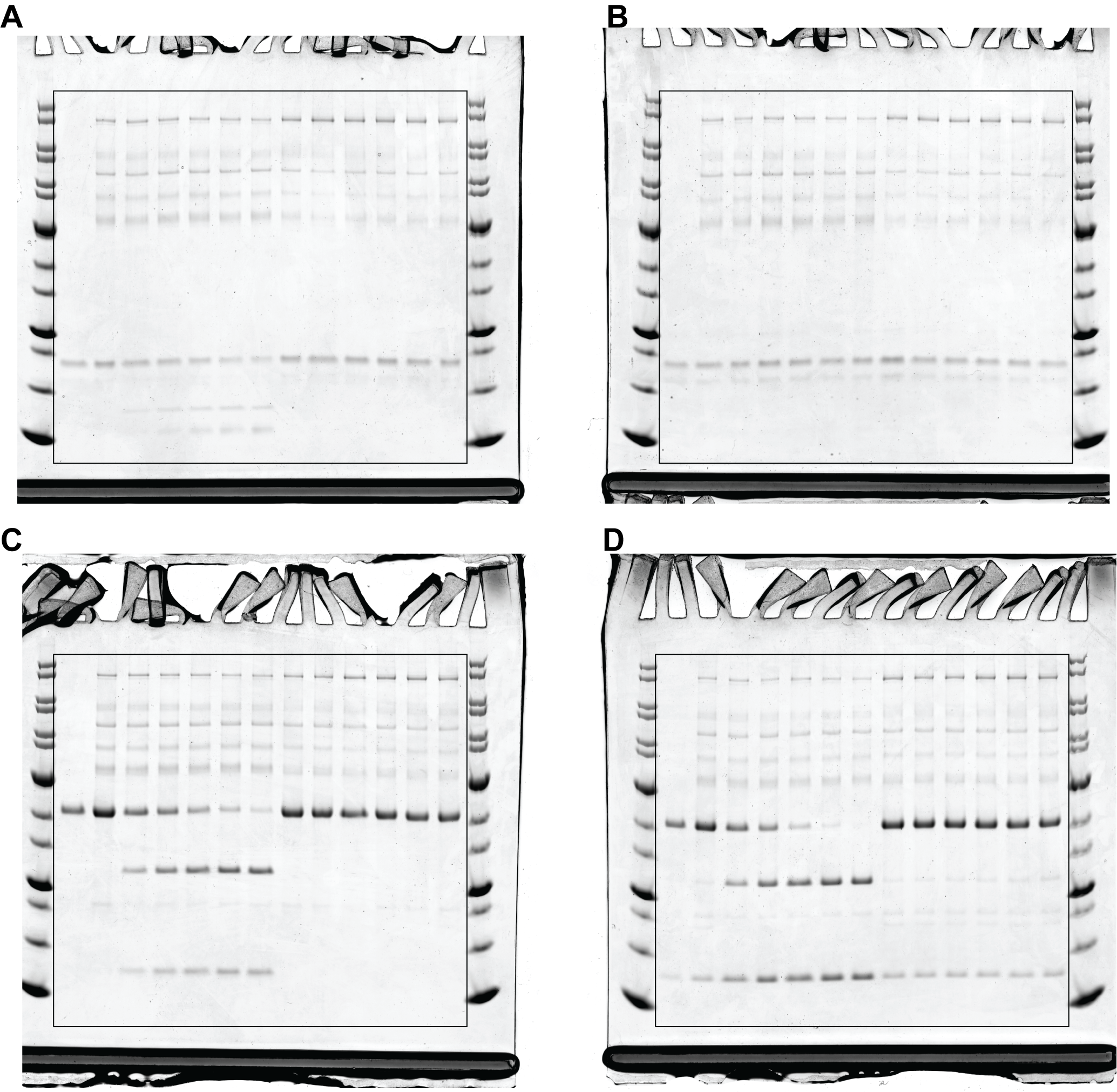

Supplement: Figure 7—source data 1. [file elife-70560-fig7-data1.png.zip › Figure7-sourcedata1.png]

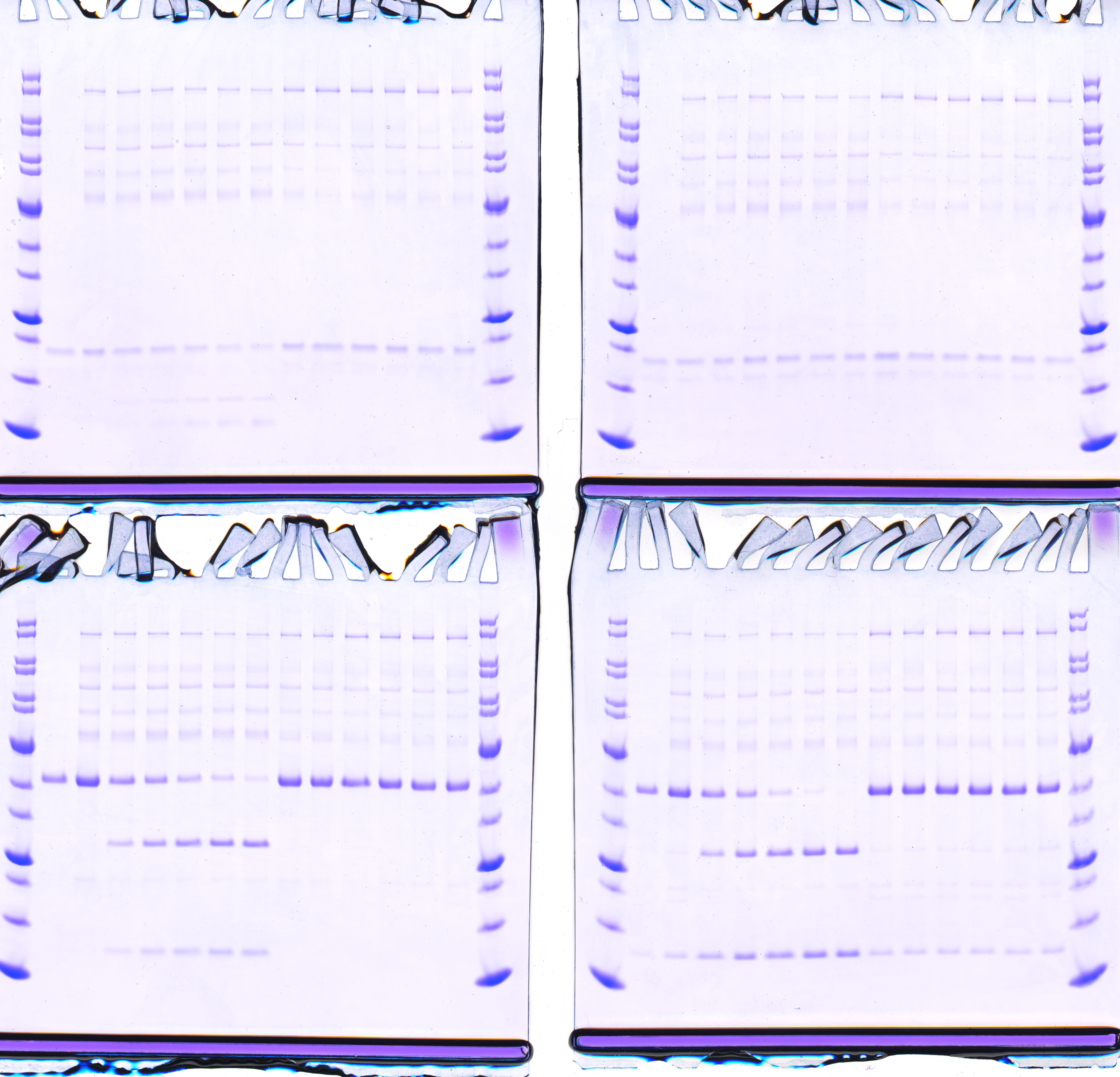

Supplement: Figure 7—source data 2. [file elife-70560-fig7-data2.tif.zip › Figure7-sourcedata2.tif]

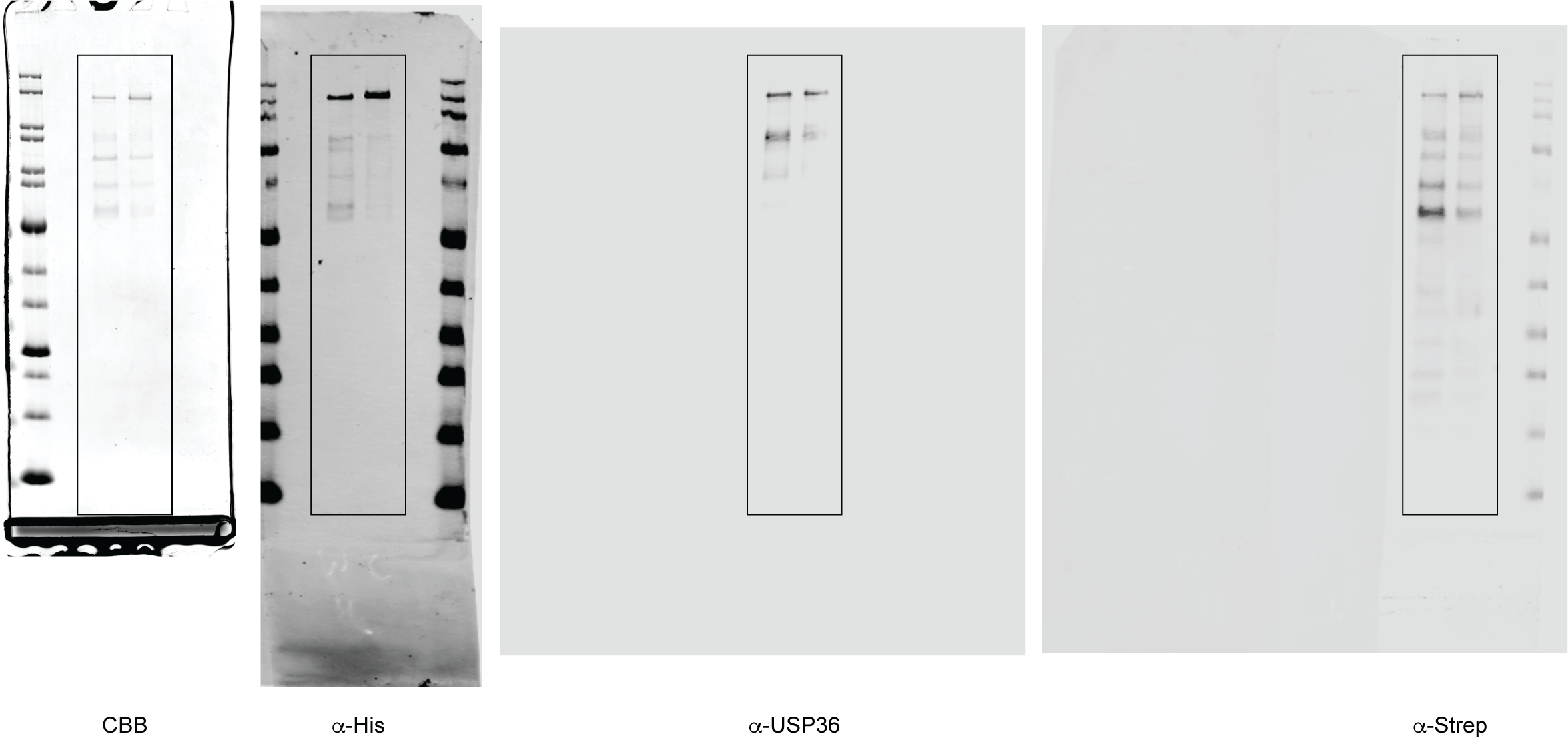

Supplement: Figure 7—figure supplement 1—source data 1. [file elife-70560-fig7-figsupp1-data1.png.zip › Figure7-figuresupplement1-sourcedata1.png]

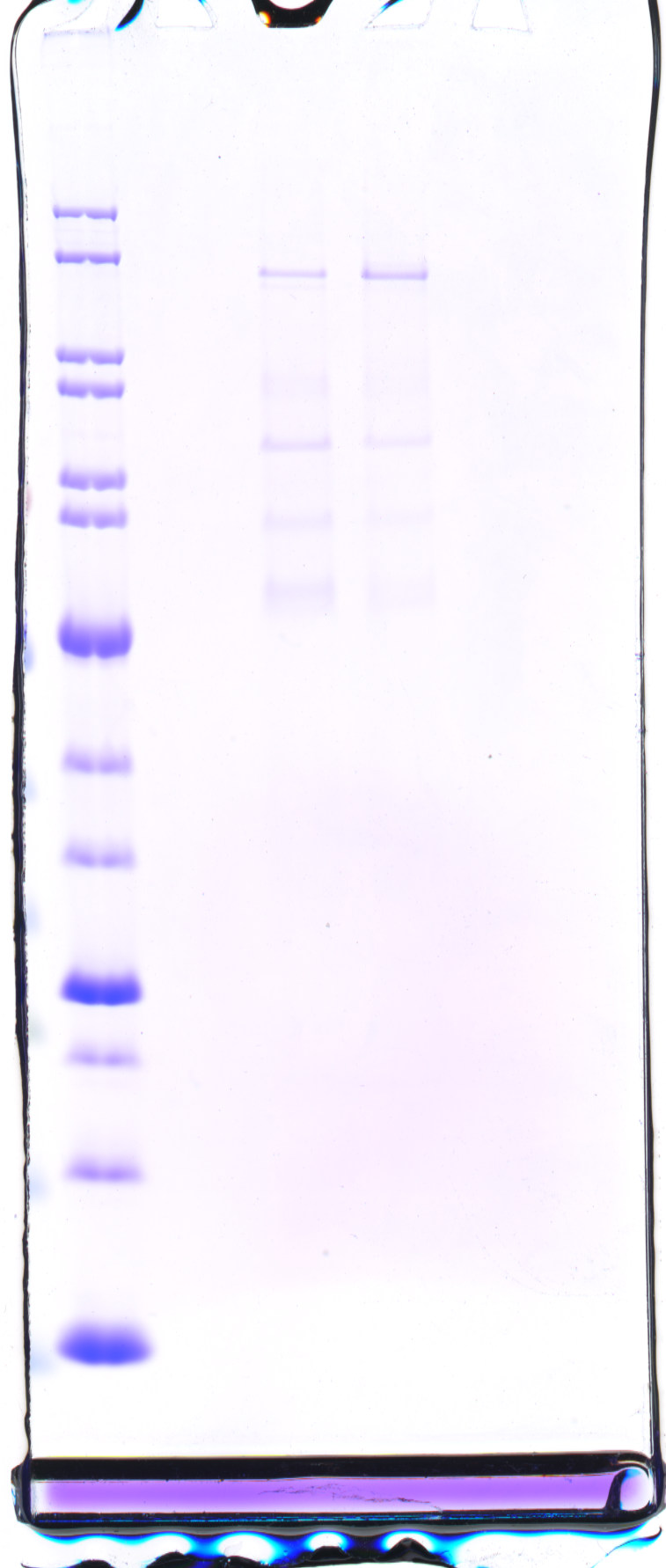

Supplement: Figure 7—figure supplement 1—source data 2. [file elife-70560-fig7-figsupp1-data2.tif.zip › Figure7-figuresupplement1-sourcedata2.tif]
