# Supplementary material for: Processing of the ribosomal ubiquitin-like fusion protein FUBI-eS30/FAU is required for 40S maturation and depends on USP36
Source: eLife. 2021 Jul 28;10:e70560. doi: 10.7554/eLife.70560 (PMC8354635; doi:10.7554/eLife.70560)
Supplement: Transparent reporting form [file elife-70560-transrepform.docx]

***eLife’s* transparent reporting form**

We encourage authors to provide detailed information *within their submission* to facilitate the interpretation and replication of experiments. Authors can upload supporting documentation to indicate the use of appropriate reporting guidelines for health-related research (see [EQUATOR Network](http://www.equator-network.org/%20)), life science research (see the [BioSharing Information Resource](https://biosharing.org/)), or the [ARRIVE guidelines](http://www.plosbiology.org/article/info:doi/10.1371/journal.pbio.1000412) for reporting work involving animal research. Where applicable, authors should refer to any relevant reporting standards documents in this form.

If you have any questions, please consult our Journal Policies and/or contact us: [editorial@elifesciences.org](mailto:editorial@elifesciences.org).

**Sample-size estimation**

- You should state whether an appropriate sample size was computed when the study was being designed
- You should state the statistical method of sample size computation and any required assumptions
- If no explicit power analysis was used, you should describe how you decided what sample (replicate) size (number) to use

Please outline where this information can be found within the submission (e.g., sections or figure legends), or explain why this information doesn’t apply to your submission:

No explicit power analysis was used to determine the sample size for each experiment. Sample size was based on empiric considerations taking into account both robustness of the respective assay and the experimental challenge of acquiring the data.

**Replicates**

- You should report how often each experiment was performed
- You should include a definition of biological versus technical replication
- The data obtained should be provided and sufficient information should be provided to indicate the number of independent biological and/or technical replicates
- If you encountered any outliers, you should describe how these were handled
- Criteria for exclusion/inclusion of data should be clearly stated
- High-throughput sequence data should be uploaded before submission, with a private link for reviewers provided (these are available from both GEO and ArrayExpress)

Please outline where this information can be found within the submission (e.g., sections or figure legends), or explain why this information doesn’t apply to your submission:

All experiments represented in the figures were performed at least in biological triplicates on independent days with the following exceptions: the experiment shown in Figure 1D was performed once with the reported experimental conditions, the experiment shown in Figure 3B was performed twice, the experiment shown in Figure 5C represents a fourth biological replicate of the experiment presented in Figures 5A and B, the experiment shown in Figure 7 was performed using the same substrate and enzyme preparations in technical triplicates on the same day, and the data shown in Figure 7–figure supplement 1 represents a single replicate. Note that while cells were induced and harvested on separate days, the StrepTactin pull-downs and MS sample preparation for Figures 5A and B were performed on the same day to minimize technical variability.

**Statistical reporting**

- Statistical analysis methods should be described and justified
- Raw data should be presented in figures whenever informative to do so (typically when N per group is less than 10)
- For each experiment, you should identify the statistical tests used, exact values of N, definitions of center, methods of multiple test correction, and dispersion and precision measures (e.g., mean, median, SD, SEM, confidence intervals; and, for the major substantive results, a measure of effect size (e.g., Pearson's r, Cohen's d)
- Report exact p-values wherever possible alongside the summary statistics and 95% confidence intervals. These should be reported for all key questions and not only when the p-value is less than 0.05.

Please outline where this information can be found within the submission (e.g., sections or figure legends), or explain why this information doesn’t apply to your submission:

Where applicable, information on the statistical tests used, number of replicates (N), definitions of center, dispersion and precision measures can be found in the respective figure legends (Figure 2B, D and F, Figure 6, Figure 2–figure supplement 1, and Figure 4–figure supplement 2D and E). P values were calculated with Prism.

For MS experiments, data analysis is described in the Materials and methods, *Affinity Purification Mass Spectrometry Analysis* and p values are reported in Supplementary file 2-2.

(For large datasets, or papers with a very large number of statistical tests, you may upload a single table file with tests, Ns, etc., with reference to sections in the manuscript.)

**Group allocation**

- Indicate how samples were allocated into experimental groups (in the case of clinical studies, please specify allocation to treatment method); if randomization was used, please also state if restricted randomization was applied
- Indicate if masking was used during group allocation, data collection and/or data analysis

Please outline where this information can be found within the submission (e.g., sections or figure legends), or explain why this information doesn’t apply to your submission:

NA

**Additional data files (“source data”)**

- We encourage you to upload relevant additional data files, such as numerical data that are represented as a graph in a figure, or as a summary table
- Where provided, these should be in the most useful format, and they can be uploaded as “Source data” files linked to a main figure or table
- Include model definition files including the full list of parameters used
- Include code used for data analysis (e.g., R, MatLab)
- Avoid stating that data files are “available upon request”

Please indicate the figures or tables for which source data files have been provided:

The raw mass spectrometry data and corresponding search results used for Figure 5B, and Supplementary file 2 have been deposited to the ProteomeXchange Consortium via the PRIDE partner repository with the dataset identifier PXD021864.
